# Floral Development of Rhamnaceae and Origin of Its Unique Floral Features

**DOI:** 10.3390/plants12020247

**Published:** 2023-01-05

**Authors:** João Paulo Basso-Alves, Carimi Cortez Ribeiro, Simone Pádua Teixeira

**Affiliations:** 1 Instituto de Pesquisas Jardim Botânico do Rio de Janeiro, Diretoria de Pesquisa Científica, Rua Pacheco Leão, 915, Rio de Janeiro 22460-030, RJ, Brazil; 2 Post-Graduate Program in Comparative Biology, Faculdade de Filosofia, Ciências e Letras de Ribeirão Preto, Universidade de São Paulo, Ribeirão Preto 14040-901, SP, Brazil; 3 Faculdade de Ciências Farmacêuticas de Ribeirão Preto, Universidade de São Paulo, Ribeirão Preto 14040-903, SP, Brazil

**Keywords:** floral ontogeny, keeled sepals, ovary position, petal and stamen development, rhamnoid, Rosales, ziziphoid

## Abstract

Rhamnaceae flowers have a peculiar morphology, including keeled sepals, one stamen whorl closely related to the petals, and a broad perigynous hypanthium that supports a voluminous nectary. In the present investigation, we detailed the flower development of five Rhamnaceae species to understand the origin of such specific floral characteristics. Floral buds and flowers were processed for surface and histological analyses. The sepals emerge in sequential order and the other organs in simultaneous order. The development of the perigynous hypanthium renders the floral apex broad and concave. The sepals undergo abaxial thickening early on, forming a keel and strongly influencing the floral merosity. Petals and stamens appear close to each other on the same radius in a very short plastochron. The carpels unite soon after their emergence, forming a syncarpous ovary and free style branches. Differences in intercalary carpel growth promote the formation of inferior (*Gouania virgata*) and semi-inferior ovaries (*Colubrina glandulosa*, *Hovenia dulcis*, and *Sarcomphalus joazeiro*). *Rhamnidium elaeocarpum* does not undergo such growth, and the resulting ovary is superior. The keeled sepals promote the isolation of the petal–stamen pair inside the flower bud. The possibility of a common primordium that the originates petal and stamen is refuted. Comparisons with other Rosales families provide insights into the floral origin and diversification of Rhamnaceae.

## 1. Introduction

The flowers of Rhamnaceae Juss. exhibit a unique combination of characters among angiosperms [1,2]. They have only antepetalous stamens [3], a relatively rare feature in few families [4]. In addition, there is always a hypanthium that supports the extra carpel whorls [5], which can form a wide disc (“disciflores”) [6] in which it is common to find a prominent intrastaminal nectary, which is quite remarkable in this family [7]. Other singularities include triangular and keeled sepals with valvate aestivation and clawed petals [1]. Some species have no petals, and others have diclinous flowers [3]. The position occupied by the ovary in the flower can vary from superior to inferior [5,8], an unusual variation within angiosperm families.

The floral characteristics of Rhamnaceae have helped to stabilize its circumscription since the earliest classifications but have also raised many doubts about its suprafamilial relationship (e.g., [6,9]). This family has been treated in a very diverse way (see [10]), i.e., as a separate order (i.e., “Rhamnales” sensu [11]) or close to families such as Vitaceae Juss. (due to antepetalous stamens, e.g., in “Rhamnales” sensu [12]), or Celastraceae R.Br. (due to the presence of a floral disc, e.g., [6,9]). The group is currently treated as a family of Rosales Bercht & J. Presl [13], which makes up the clade of Pentapetalae D.E. Soltis, P.S. Soltis & W.S. Judd [14]. Flowers are predominantly pentamerous in this family, and the gynoecium usually has a small merosity— approximately (1)2–4 carpels [5]. Rosales comprise Rosaceae Juss. as a sister group of a lineage with two main clades, with Rhamnaceae and three other families (Barbeyaceae Rendle, Dirachmaceae Hutch., and Elaeagnaceae Juss.) assigned to one of them and urticalean rosids (Cannabaceae Martinov, Moraceae Juss., Ulmaceae Mirb., Urticaceae Juss.) to the other—formerly treated as “Urticales” [15,16]. The relationship of Rhamnaceae with other families in its clade remains controversial [15,16,17].

Rosales flowers can have a hypanthium with nectary, valvate calyx, clawed petals, and one ovule per carpel [18]. In Rosaceae, the flowers have a conspicuous perigynous hypanthium and a diplo- or polystemonous androecium, with variation in ovary position [2,19]. Urticalean rosids have apetalous flowers, usually diclinous and with no hypanthium, with antesepalous stamens and a pseudomonomerous gynoecium [2,20]. The families closest to Rhamnaceae show some floral characteristics distinct from these [21]. Barbeyaceae differ from Rhamnaceae in having no hypanthium or nectaries and more stamens, although their position, whether antepetalous or antesepalous, is uncertain. Dirachmaceae, like Rhamnaceae, has only antepetalous stamens but a larger perianth merosity (5–8) and a contorted corolla. Elaeagnaceae lacks a corolla, and its androecium is diplostemonous in the members of *Shepherdia* Nutt. but not in *Elaeagnus* L. and *Hippophae* L. Thus, the hypanthium is variably developed or absent, at least within the Barbeyaceae and urticalean rosids.

Floral characteristics are also important for the internal circumscription of Rhamnaceae, in which the ovary position appears as the most important characteristic [5,8]. This family is cosmopolitan, comprises approximately 900 species belonging to 60 genera [5,22], and is composed of three main lineages: “ziziphoids”, with 37 genera, a sister group of the lineage comprising “ampelozizyphoids” (3 genera) + “rhamnoids” (20 genera) [22,23,24]. Superior ovaries and drupaceous fruits are found predominantly in rhamnoids and inferior ovaries, and schizocarpic fruits are found in ziziphoids [23]. The three main lineages have representatives with semi-inferior ovaries. Currently, 11 tribes are recognized for the family [23], but several genera have dubious relationships or have not been formally assigned to any of these tribes (*incertae sedis*), such as *Ceanothus* L., *Colubrina* Rich. ex Brongn., and *Sarcomphalus* P. Browne (see [24,25]).

Thus, the objective of the present investigation was to study the developing flowers of five Rhamnaceae species in order to understand peculiar floral structural issues, such as (a) the formation of inward keeled sepals; (b) the ontogenetic origin of the antepetalous stamens, considering that previous studies have suggested that the stamens and petals originate from a common primordium [3], with important implications for plastochron evolution [26]; and (c) variation in the ovary position [8,27,28]. This type of variation is associated with a particular type of hypanthium (sensu [29]), the gynoecial hypanthium, forming inferior ovaries. This contrasts the perigynous hypanthium, which is not associated with the ovary.

## 2. Results

### 2.1. Organography

The flowers are small (about 1–5 mm long) and generally pentamerous but with variations between tetramerous (*Hovenia dulcis*) and hexamerous types (*Colubrina glandulosa*, *Sarcomphalus joazeiro*), monoclinous, polysymmetric, and dichlamydeous. A perigynous hypanthium is present, usually campanulate, with a larger opening in some species than in others. The calyx has valvate aestivation and is composed of free, coriaceous, triangular sepals with the median nervature prominent on the abaxial surface, forming a keel, usually with a callous apex. The corolla is made up of tiny petals that never touch each other and that are clawed, with a cucullate or concave apex. The androecium is formed by a single whorl of stamens opposite to the petals and joined to them by the base of the filaments. The anthers are bithecal, tetrasporangiate, and introrse and have longitudinal dehiscence and dorsifixed insertion in the filament. The gynoecium varies in carpel number between species: one carpel in *Rhamnidium elaeocarpum*, two to three in *Colubrina glandulosa* and *Hovenia dulcis*, three in *Gouania virgata*, and two to four in *Sarcomphalus joazeiro*. The position of the ovary also varies according to the species: inferior in *Gouania virgata*; semi-inferior in *Colubrina glandulosa*, *Hovenia dulcis*, and *Sarcomphalus joazeiro*; and superior in *Rhamnidium elaeocarpum*. The distal end of the style may show varying degrees of free stigmatic branches. There is one erect anatropous ovule per locule, with basal placentation. Intrastaminal nectaries occur in all species and may form a conspicuous disc (*Colubrina glandulosa*) or not (*Hovenia dulcis*). This nectary can also be restricted to the perigynous hypanthium (*Rhamnidium elaeocarpum* and *Sarcomphalus joazeiro*) or extend over part of the ovary roof (*Gouania virgata*).

### 2.2. Ontogeny

**Figure 1:**
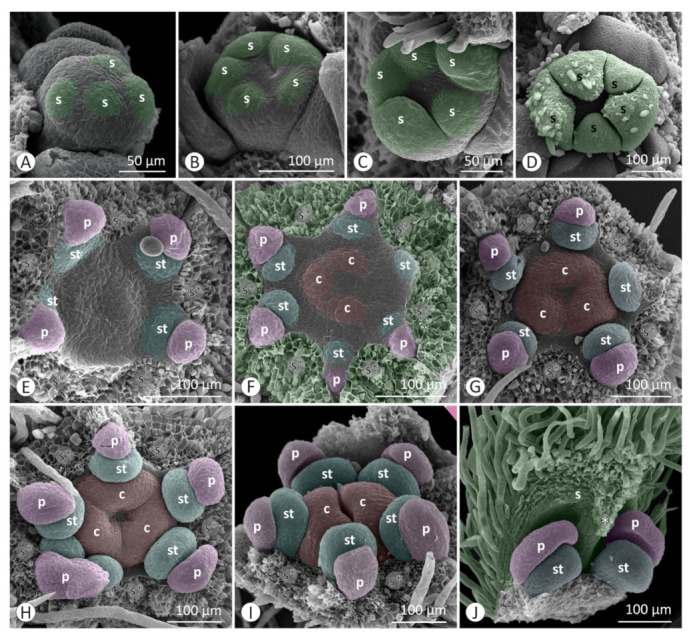
Floral development of *Colubrina glandulosa*. (**A**–**J**) = SEM. (**A**) Emergence of sepal primordia at the floral apex. (**B**) Floral apex flanked by two lateral prophylls and a helical sequence of sepal emergence. (**C**) Elongation of the young sepals, especially the abaxial median, over the rest of the flower bud. (**D**) Valvate aestivation of young sepals and non-secretory trichomes developing on the outer surface of the sepals. (**E**) Slightly elevated region where petals and stamens emerge, in contrast to the flat interior of the flower bud. Emergence of the stamen primordia after the primordia of petals. (**F**) Hexagonal contour (delimited by the sepals (s*)) of the rest of the floral apex and primordia of petals, stamens, and carpels. (**G**) Lateral union between three young carpels. (**H**) Closure of the carpel cleft in the plicate portion of young carpels. (**I**) Young petals are approximately the same size as young stamens and form a flattened structure. (**J**) The young sepal exhibits a median thickening that projects towards the interior of the flower bud (*). The young petals and stamens occupy the flanks of the sepals. Symbols and colors: c = carpel (red), p = petal (pink), s = sepal (green), st = stamen (blue), * = keel/apex of sepal, s* = sepal removed.

**Figure 2:**
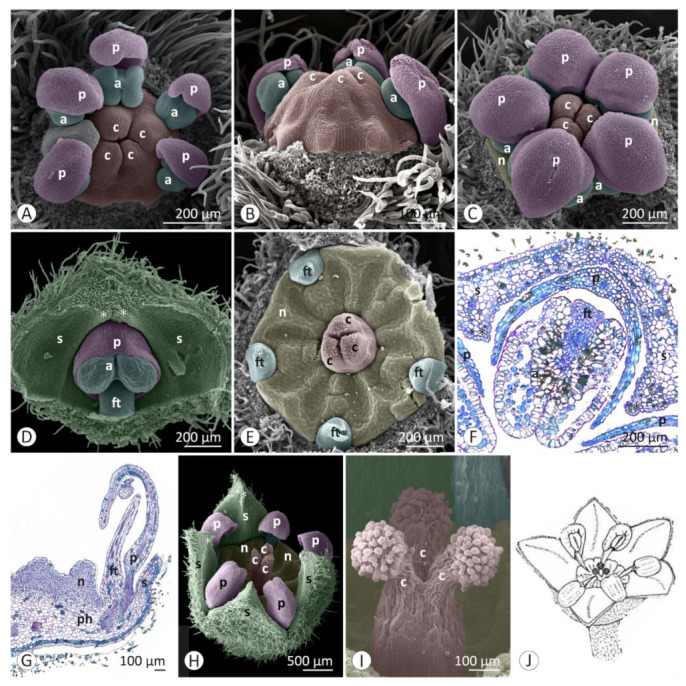
Floral development of *Colubrina glandulosa*: (**A**–**E**,**H**,**I**) SEM; (**F**,**G**) LM. (**A**) Young bud with petals surrounding the stamens. Differentiating anthers with two thecae arranged opposite each other and joined by the connective. The lateral union between carpels does not reach the most distal region of the gynoecium. (**B**) Young petals and stamens slightly inclined over the gynoecium and developing nectary. (**C**) Flower bud with cucullate petals over the stamens and stigmatic apex of the gynoecium. (**D**) Young stamen covered by the young petal. These structures grow between two adjacent sepals in the gap delimited by the median projection of the sepals. (**E**) Formation of the nectariferous disk around the gynoecium. Marks on the disk show the contour of the anthers, which maintain contact with the disk surface. (**F**) A petal covers the stamen and valvate prefloration of the sepals (cross section). (**G**) Insertion of the sepal, petal, and stamen on the distal margin of the perigynous hypanthium (longitudinal section). (**H**) Bud at the beginning of anthesis, with anthers enveloped by cucullate petals. (**I**) Stigmatic surface over the trifid end of the style. (**J**) Illustration of a flower at anthesis. Symbols and colors: a = anther (blue), c = carpel (red), ft = filament (blue), n = nectary (yellow), p = petal (pink), ph = perigynous hypanthium, s = sepal (green), * = keel/apex of sepal.

**Figure 3:**
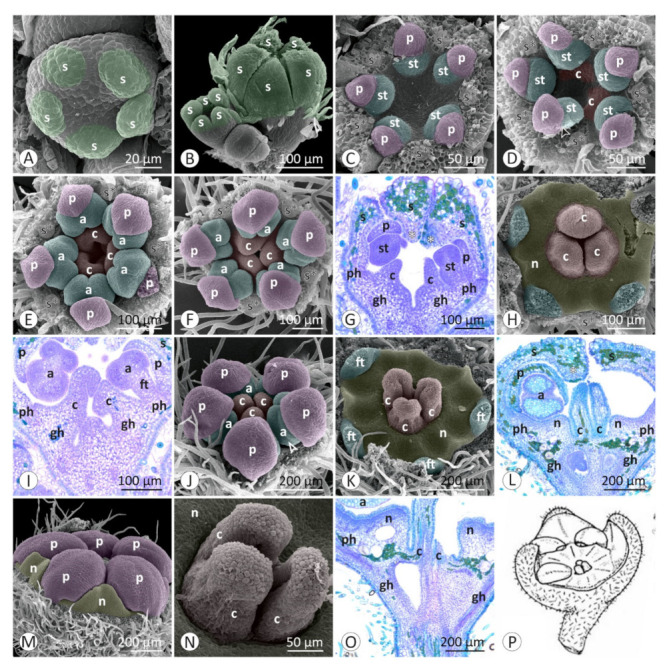
Floral development of *Gouania virgata*: (**A**–**F**,**H**,**J**,**K**,**M**,**N**) SEM; (**G**,**I**,**L**,**O**) LM. (**A**) Helical emergence of the sepals. (**B**) Sepal growth and valvate aestivation calyx. (**C**) Slightly concave interior of the flower bud, particularly in the region where petals and stamens emerge. (**D**) Emergence of carpels and basal union between petals and stamens (arrow). (**E**) Laminar young petals, differentiation of thecae in the anther, and carpel cleft formation. (**F**) Carpel apices are close. (**G**) Massive median region of sepals (*) and differentiation of the stamen into anther and filament; young carpels with a free distal region and basal region associated with the gynoecial hypanthium (longitudinal section). (**H**) Nectariferous disk formation and free young styles. (**I**) Closure of the carpel cleft and ovary locule delimitation (longitudinal section). (**J**) Young petals cucullate over stamens; differentiation of thecae in pollen sacs (arrow). (**K**) Stigmatic differentiation on plicated branches of the styles. Marks on the nectariferous disk reveal the contact of this surface with the anthers. (**L**) The anthers are leaning over the nectariferous disk and are covered by the petals. The nectariferous disk is also elevated over the roof of the inferior ovary and free styles (longitudinal section). (**M**) The nectariferous disk forms peripheral laminar projections that rise between the petals. (**N**) Stigmatic surface on free style branches in pre-anthesis. Grooves are present in the plicate region of the carpels. (**O**) Inferior ovary during pre-anthesis (longitudinal section). (**P**) Illustration of a flower at pre-anthesis. Symbols and colors: a = anther, c = carpel (red), ft = filament, gh = gynoecial hypanthium, n = nectary (yellow), p = petal (pink), ph = perigynous hypanthium, s = sepal (green), st = stamen (blue), * = keel/apex of sepal, s* = sepal removed.

**Figure 4:**
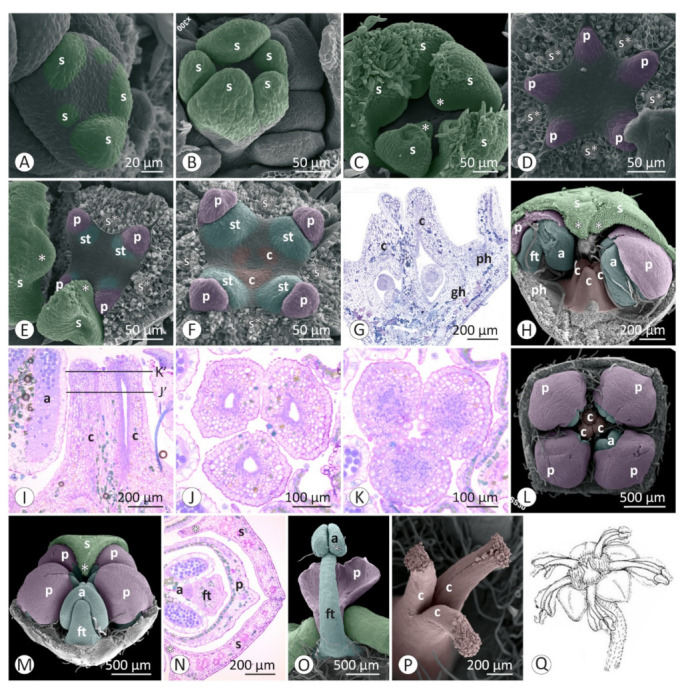
Floral development of *Hovenia dulcis*: (**A**–**F**,**H**,**L**,**M**,**O**,**P**) SEM; (**G**,**I**–**K**,**N**) LM. (**A**) Helical emergence of the sepals. (**B**) Growth of young sepals around the flat floral apex. (**C**) Calyx with valvate aestivation and formation of non-glandular trichomes on the outer surface of the sepals. The median region of the young sepals grows towards the interior of the flower bud, forming a keel-shaped projection. (**D**) Interior of the floral apex with petal primordia emerging at the vertices of the pentagonal space delimited by sepals (s*). (**E**) Emergence of stamen primordia internal to the petal primordia. The keel of the sepal is in contact with the inner surface of the floral apex. (**F**) Quadrangular outline of the floral apex, slightly concave, with young petals, primordia of stamens, and carpels. (**G**) Semi-inferior ovary with two visible locules. Incomplete closure of the distal portion of the young gynoecium (longitudinal section). (**H**) Young stamens inclined over the gynoecium, expanding perigynous hypanthium, cucullate petals, and valvate aestivation sepals. (**I**) Style with free distal regions (longitudinal section). (**J**) Three style branches with hollow transmitting tissue (cross section from the proximal region (J’) indicated in (**I**)). (**K**) Three style branches close to the stigma (cross section from the distal region (K’) indicated in (**I**)). (**L**) Tetramerous flower bud with cucullated petals covering the stamens. (**M**) Pentamerous flower bud with an exposed stamen showing dorsifixed insertion of the anther into the connective. (**N**) Stamen covered by a petal and valvate aestivation of the sepals. (**O**) Stamen and associated petal, both reflexed during anthesis. (**P**) Trifid end of the style with distinct stigmas on each branch. (**Q**) Illustration of an anthetic flower. Symbols and colors: a = anther, c = carpel (red), ft = filament, gh = gynoecial hypanthium, p = petal (pink), ph = perigynous hypanthium, s = sepal (green), st = stamen (blue), * = keel/apex of sepal, s* = sepal removed.

**Figure 5:**
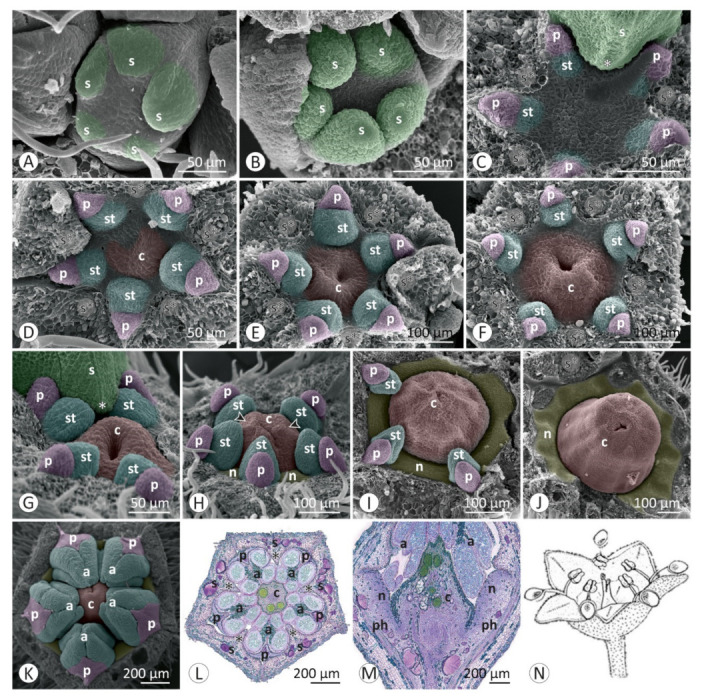
Floral development of *Rhamnidium elaeocarpum*: (**A**–**K**) SEM; (**L**,**M**) LM. (**A**) Helical emergence of the sepals. (**B**) Young sepals and flat interior of the floral apex. (**C**) Emergence of petals and stamens on the slightly flat surface of the floral apex. The pentagonal contour is delimited by the young sepals, the median region of which forms a keel-shaped projection over the interior of the floral bud. (**D**) Emergence of the carpel primordium. Stamen primordia become larger than petal primordia. (**E**) Formation of the carpel cleft. (**F**) Elongation of the young carpel. (**G**) The region of insertion of the petals and stamens is relatively flat in relation to the rest of the floral apex. The young sepal grows towards the internal floral organs to such an extent that the median projection is in contact with petals and stamens at this stage. (**H**) Young stamens become flattened and grow more than the petals. Nectary formation occurs in the space between the stamens and the carpel; the keeled sepal over the gynoecium is marked by arrows. (**I**) Young petals and stamens slightly curved towards the gynoecium. (**J**) Distal closure of the carpel cleft. (**K**) Pre-anthesis pentamerous bud with petals that are shorter than the stamens. (**L**) Abaxial pollen sacs larger than adaxial pollen sacs. Petals cover a small portion on the abaxial surface of stamens. The keel on the inner surface of the sepal grows between adjacent anthers (cross section). (**M**) Superior ovary and perigynous hypanthium with a nectariferous disk (longitudinal section). (**N**) Illustration of an anthetic flower. Symbols and colors: a = anther, c = carpel (red), n = nectary (yellow), p = petal (pink), ph = perigynous hypanthium, s = sepal (green), st = stamen (blue), * = keel/apex of sepal, s* = sepal removed.

**Figure 6:**
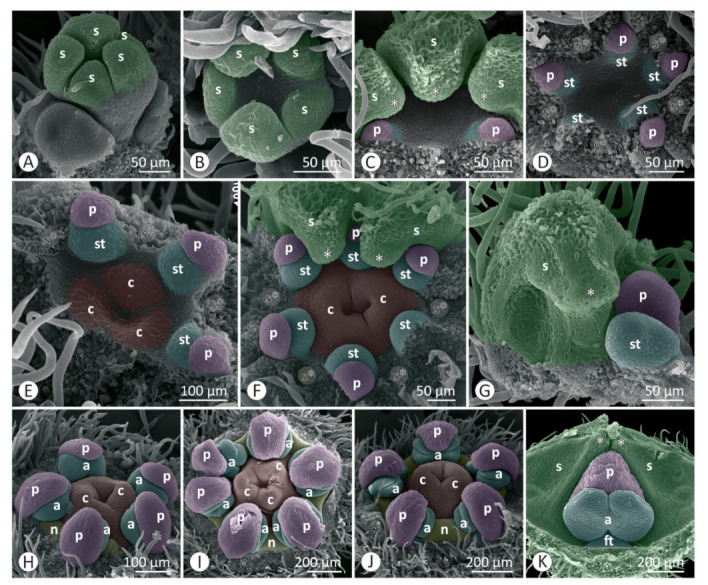
Floral development of *Sarcomphalus joazeiro*: (**A**–**K**) SEM. (**A**) Floral apex before sepal emergence (below) and a floral bud with young sepals in valvate aestivation (above). (**B**) Growth of young sepals in the flower bud. (**C**) Emergence of the petal and stamen primordia opposite the young sepals. The inner surface of the floral apex is relatively flat, and the sepals form a keeled projection towards the center of the floral bud. (**D**) Emergence of stamen primordia after petal primordia. (**E**) Emergence of carpel primordia and elevation of petal and stamen primordia. (**F**) Carpel cleft formation in two young plicated carpels joined at the base. (**G**) Young sepal with adaxial keel and young petal and stamen housed on the sepal flank. (**H**) Young petals become laminar, with theca differentiation (one opposite the other) in the anther. (**I**) Twinned petals and stamens are slightly shorter than the others. (**J**) Young stamens slightly inclined towards the developing nectary and differentiation of pollen sacs in the anther. (**K**) Stamen partially covered by the petal, forming a unit arranged between two sepals. Symbols and colors: a = anther, c = carpel (red), ft = filament, n = nectary (yellow), p = petal (pink), s = sepal (green), st = stamen (blue), * = keel/apex of sepal, s* = sepal removed.

**Figure 7:**
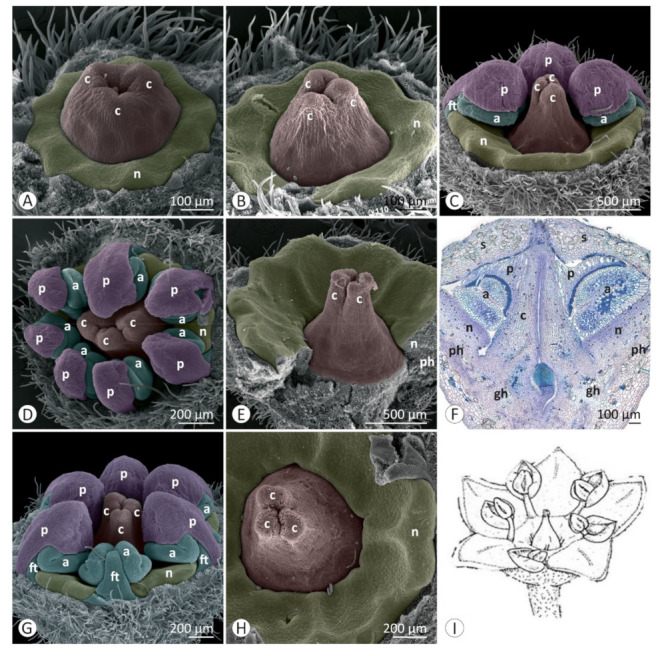
Floral development of *Sarcomphalus joazeiro*: (**A**–**E**,**G**,**H**) SEM; (**F**) LM. (**A**) Elongation of laterally united young carpels. The region of contact with stamens can be seen in the nectary and gynoecium (arrows). (**B**) Later growth of the gynoecium as shown in (**A**) and elongation of style branches. (**C**) Cucullate young petals cover the stamens; this set remains leaning over the nectariferous disk during pre-anthesis. (**D**) Atypical flower bud with seven petals and seven stamens. (**E**) The nectariferous disc over the perigynous hypanthium does not extend over the gynoecium. (**F**) Semi-inferior ovary and hollow style (longitudinal section). (**G**) Clawed and cucullate petals on stamens. Union between the short basal regions of the filament and petal. (**H**) Gynoecium with the style distally divided into three short branches and continuous stigma. (**I**) Illustration of an anthetic flower. Symbols and colors: a = anther, c = carpel (red), ft = filament, gh = gynoecial hypanthium, n = nectary (yellow), p = petal (pink), ph = perigynous hypanthium, s = sepal.

Floral developmental stages were described and illustrated for *Colubrina glandulosa* (Figure 1 and Figure 2), *Gouania virgata* (Figure 3), *Hovenia dulcis* (Figure 4), *Rhamnidium elaeocarpum* (Figure 5), and *Sarcomphalus joazeiro* (Figure 6 and Figure 7).

The sepals emerge from the floral apex as distinct primordia in sequential order (*Colubrina glandulosa* (Figure 1A–C), *Gouania virgata* (Figure 3A,B), *Hovenia dulcis* (Figure 4A,B), *Rhamnidium elaeocarpum* (Figure 5A,B), and *Sarcomphalus joazeiro* (Figure 6A,B)). Shortly afterwards, the young sepals form a medial thickening along their entire adaxial extension, culminating in an apical callous projection. After the initial elongation that defines the valvate calyx, they circumscribe the remaining space in the floral apex for the emergence of the other floral organs. This space is usually star-shaped, with the sides arched inwards, like a hypocycloid. The shape of this “star” varies according to the number of sepals, with five points (most common; Figure 1G, Figure 3C,D, Figure 4D, Figure 5C–F and Figure 6D), four points (astroid-like; Figure 4E,F), or six points (Figure 1F and Figure 6F). The arching of this hypocycloid is due to the medial thickening of the developing sepals. The primordia of petals and stamens emerge alternately with the sepals in the position corresponding to the vertices of this star. This region can be elevated by the growth of the perigynous hypanthium to a greater or lesser extent depending on the species. However, in general, the inner surface of the floral apex is relatively broad and flat.

The petals emerge in simultaneous order (Figure 4D) and are followed by the antepetalous stamens (Figure 1E,F, Figure 4E,F, Figure 5C,D and Figure 6C,D), with a short plastochron between the two organ types. Although petals and stamens appear as distinct primordia, they are united at the base, even in the early stages, and there is a common basal growth of these primordia, as in *Gouania virgata* (Figure 3D) and *Rhamnidium elaeocarpum* (Figure 5D). Then, the carpel primordia emerge (Figure 1F,G, Figure 3D, Figure 4F, Figure 5D and Figure 6E) and may vary in number according to the species or even within the same species. In *Rhamnidium elaeocarpum*, there is only one carpel primordium (Figure 5E–K), whereas in *Gouania virgata* (Figure 3E) and *Hovenia dulcis*, there are three carpel primordia (Figure 4H,J). *Sarcomphalus joazeiro* may have two (Figure 6F,H,J) or three carpel primordia (Figure 1G,H and Figure 6I), whereas *Colubrina glandulosa* may have two (Figure 1I), three (Figure 1G,H), or four carpel primordia (Figure 2A).

The inner keeled projection of young sepals delimits locule-like spaces in which young petals and stamens grow (Figure 1J, Figure 2D, Figure 4E and Figure 6G,K). In this stage, non-glandular trichomes form on the outer surface of the sepals, as in *Colubrina glandulosa* (Figure 1D,J), *Hovenia dulcis* (Figure 4C), and *Sarcomphalus joazeiro* (Figure 6C,G).

Although the petals emerge a little earlier than the stamens, they grow less than the stamens during the intermediate stages. Young stamens become flattened (Figure 1H–J, Figure 5H and Figure 6G) on the abaxial–adaxial plane, and soon differentiate into anther and filament (Figure 3E and Figure 6H). The anthers usually have two thecae on opposite sides of this flattened structure (Figure 2A, Figure 3E,F and Figure 6H). Shortly thereafter, it is possible to observe the formation of furrows that delineate the limit between the pollen sacs of the same theca (Figure 2B, Figure 3F,J, Figure 5I and Figure 6I,J). Petals may form cucullate structures that cover most of the stamens or only their abaxial surface (e.g., *Rhamnidium elaeocarpum*; Figure 5I,K). In *Sarcomphalus joazeiro*, the petals form clawed structures in which the lamina surrounds part of the anther, whereas the claw accompanies the filament. The basal regions of the petal and filament are united at the base (Figure 7G).

The carpel primordia emerge on a relatively flat surface in the center of the floral apex (Figure 1E–G, Figure 3C,D, Figure 4E,F, Figure 5C–F and Figure 6C–F). The young carpels form plicate structures and are laterally united at the base in the case of a syncarpous gynoecium. The carpel cleft has an acropetal closure. The lateral union between carpels can vary greatly in relation to their distal end. The style branches can be very short (*Colubrina glandulosa* and *Sarcomphalus joazeiro*), short (*Hovenia dulcis*), or long (*Gouania virgata*). In the case of *Gouania virgata*, each carpel exhibits a style free from the others throughout its extension. The formation of semi-inferior ovaries results from the growth of the gynoecial hypanthium in varying degrees: smaller in *Sarcomphalus joazeiro* (Figure 7F) than in *Colubrina glandulosa* or *Hovenia dulcis*. In *Gouania virgata*, the growth of the gynoecial hypanthium is conspicuous before the closing of the carpel cleft, leading to the formation of a completely inferior ovary (Figure 3I,L,O).

The expansion of the perigynous hypanthium causes both an increase in floral diameter and an elevation of the perianth and androecium organs. This phenomenon coincides with the formation of the nectariferous disk over the perigynous hypanthium. The valvate closure of the calyx, which is associated with the formation of a discoid perigynous hypanthium, i.e., a structure that is more flat than deep, causes stamens and petals to bend over the developing nectariferous disk (Figure 1C, Figure 3L, Figure 4H, Figure 5I, Figure 6J and Figure 7C,F,G). As a result, we often observe marks of this contact on the nectariferous surface; the anthers are pushed against the nectary, which, in turn, expands in regions less subject to mechanical pressure, forming molds around the anthers (Figure 2E, Figure 3K and Figure 7A–C,E,H). Such marks are no longer evident during nectary development, but the contour of this structure at anthesis may retain some impressions (Figure 2G,H). During anthesis, after calyx opening, the unit formed by the petal and stamen moves from a curved position on the flower bud (Figure 7G) to an erect position (Figure 2H) and is finally reflexed (Figure 4O) to the floral axis.

In *Sarcomphalus joazeiro*, some flower buds showed two petals and two stamens in the apex region delimited by the sepals (Figure 6I). These petals and stamens are slightly smaller than the others. Some buds have only one vertex with twinned organs (Figure 6I), whereas others have more than one region, leading to the formation of a bud with seven petals and seven stamens (Figure 7D).

## 3. Discussion

### 3.1. Calyx: Development and Implications for the Floral Construction

Among all the peculiar floral characteristics of the studied Rhamnaceae species, the keeled sepals, i.e., sepals endowed with a medial thickening on the inner surface, stand out because they represent an uncommon condition not only in the family but also among angiosperms. Although many angiosperms have carinate organs in the perianth, especially between the outer whorls, such carinae are often abaxial, as in some orchids [30] or *Passiflora* L. [31]. An interesting case occurs in the genus *Coriaria* Niss. ex L. (Coriariaceae DC., Cucurbitales Juss. ex Bercht. & J. Presl), in which the internal carina of the fleshy petals grows between the carpels, giving the compound fruit a berry-like appearance [32]. In Rhamnaceae, the degree of prominence of this keel may vary, even within the same genus, as in species of *Reynosia* Griseb. [33].

The existence of keeled sepals in Rhamnaceae promotes several effects on their floral construction. The development of an internal keel creates a compartmentalization of the inner space of the flower bud. The petals and stamens, formed in an alternating position between the sepals, remain isolated by “locules” between adjacent sepals. This effect seems to be quite pronounced during development in *Colubrina glandulosa* and *Sarcomphalus joazeiro* and may have drastic implications for the development of internal organs, especially regarding floral merosity, as well as anthesis dynamics.

An important issue is the shape of the sepal in relation to the rest of the developing floral apex. Unlike laminar sepals, which delimit a simple geometric figure (e.g., a pentagon), the keeled sepals of Rhamnaceae delineate a more complex figure (e.g., a five-pointed star) and occupy a large portion between the vertices of the contour of the floral apex. It is interesting to note that there is not even a trace of the emergence of antesepalous stamens in Rhamnaceae.

Another issue is that we found five sepals in most of the flowers sampled, with the occasional occurrence of buds with four or six sepals. The emerging petals and stamens alternate with the growing sepals [3,6,27,31]. Thus, the number of sepals defines the shape of the inner space of the floral apex, where the subsequent organs will emerge, i.e., petals, stamens, and carpels. Regardless of the number of sides of this star (tetra-, penta-, or hexagonal), the petals and stamens always emerge at the vertex, following the alternate position of the sepals. In this context, the number of developing sepals seems to determine the corolla and androecium merosity in Rhamnaceae flowers. Thus, this family is an illustrative example of how the merosity of the outer whorls influences the merosity of the inner whorls (e.g., [34]).

The sepals of Rhamnaceae appear as independent organs and in sequential order (present study), as previously verified [31,35], although the emergence order can also be “almost simultaneous” in some species (e.g., [27]). Thus, the development of the sepals in Rhamnaceae culminates in a strongly cohesive valvate aestivation calyx in pre-anthesis. This type of calyx aestivation is quite common [2].

Finally, the arrangement of the keeled sepals seems to cause the petals and stamens to remain strongly curved inside the flower bud, delaying filament elongation and petal expansion until the moment the calyx opens.

### 3.2. Hypanthia and Gynoecium

All studied Rhamnaceae species have a perigynous hypanthium, which can vary greatly in its morphology from a broad, flat floral disk to a long tube (e.g., Colletieae Reiss. ex Endl.) [5]. The formation of a perigynous hypanthium can be observed from the establishment of a flat floral apex, the edges of which are raised by a marginal growth resulting from the activity of intercalary meristems (sensu [29]). In this sense, the radial growth of the perigynous hypanthium is an important marker of the formation of a disk flower, which is typical of Rhamnaceae (see [36]). The development of the perigynous hypanthium is quite conserved in this family, as reported herein and in previous studies [3,6,27,37]. One factor that seems to vary among species is the direction of this intercalary growth, in the sense of promoting the formation of a flat (disk-shaped, e.g., *Sageretia* Brongn., *Ziziphus* Mill.), a campanulate (e.g., *Gouania* Jacq., *Helinus* E.Mey. ex Endl., *Lasiodiscus* Hook.f., *Nesiota* Hook.f., *Reynosia*, *Rhamnus* L., and *Trevoa* Miers), or a tubular perigynous hypanthium (e.g., *Colletia* Comm. ex Juss., *Cryptandra* Sm., *Discaria* Hook., *Kentrothamnus* Suess. & Overkott, and *Retanilla* (DC.) Brongn.) [5]. Such floral constructions are functionally dissimilar, especially in terms of accessibility to resource-seeking floral visitors, but are formed in basically the same way. In the case of Rhamnaceae, the main alteration of the perigynous hypanthium is related to the floral nectary, one of the most conspicuous floral features of the family. The structured nectary of Rhamnaceae originates on the peripheral portion of the carpels and expands in diameter as it grows, along with the length of the perigynous hypanthium [7]. Although the structural characteristics of nectaries can vary greatly in this family, their mode of development is very similar between species [7].

Importantly, the mere existence of intercalary meristems is not sufficient to explain floral variation in Rhamnaceae. The degree of concavity of the floral apex during the early stages of development has already been credited as a predictor of the position that the ovary will occupy in the structure at anthesis.

In this sense, flat apices form superior ovaries, e.g., *Oreoherzogia pumila* (Turra) W.Vent (=“*Rhamnus pumilus*”) [31] and *Rhamnus* [3], and concave apices culminate in inferior ovaries, e.g., *Gouania virgata* (present study), or semi-inferior ovaries, e.g., *Hovenia dulcis* (present study). Such an association has been proposed for Rhamnaceae in particular (e.g., [8]) and other families of angiosperms in general (e.g., [38]). However, this correlation does not seem to be valid for several cases, such as Melastomataceae Juss. [39] or Rosaceae [40]. In Rhamnaceae, more flattened floral apices may form semi-inferior ovaries (e.g., *Colubrina glandulosa*, *Paliurus spina-christi* Mill. [3], and *Sarcomphalus mauritianus* (Lam.) Raf. (=*Ziziphus mauritiana* Lam.) [35]), and concave apices may form superior ovaries (e.g., *Rhamnidium elaeocarpum*). Thus, other factors interfere with the degree of inclination of the floral apex and its transformation during development in Rhamnaceae. In this sense, it is important to understand how the dynamics of intercalary growth occurs during floral development in Rhamnaceae.

The species studied here illustrate how the ovary can vary from superior to inferior in Rhamnaceae. This variation in ovary position results from the action of a particular type of intercalary growth associated with the carpel, which culminates in the formation of the gynoecial hypanthium. The onset of a perigynous hypanthium usually precedes that of a gynoecial hypanthium. A gynoecial hypanthium is not always present in Rhamnaceae flowers, unlike the perigynous hypanthium. The greater the intensity of intercalary growth associated with the carpel, the greater the inferior portion of the ovary, as demonstrated by our results (formation of inferior ovaries in *Gouania virgata*, semi-inferior ovaries in *Hovenia dulcis* and *Sarcomphalus joazeiro*, and superior ovaries in *Rhamnidium elaeocarpum*). Thus, the position of intercalary meristems and the duration and direction of growth are fundamental for floral diversification, explaining the variation in ovary position in Rhamnaceae.

From a taxonomic point of view, the main groups of Rhamnaceae—ampelozizyphoids, rhamnoids, and ziziphoids—have genera with semi-inferior ovaries and their variations [5,23,25]. This suggests a very high lability in ovary position in the family. Thus, plasticity in the development of the gynoecial hypanthium must be an inherent feature of Rhamnaceae. Ovary position tends to be a stable characteristic within angiosperm families, but several groups show similar disparity, such as Melastomataceae [39] and Saxifragaceae [41]. However, in rhamnoids, several genera have a superior ovary [5], and there are few cases of an inferior ovary (e.g., *Fenghwaia* [22]). The opposite situation is found among ziziphoids, in which inferior ovaries are frequent (especially among Gouanieae Reissek ex End. and Phyliceae Reissek ex Endl.), and there are few genera with superior ovary species (e.g., *Blackallia* C.A.Gardner and *Sarcomphalus*, *Zizyphus* s.s.) [5,25].

The two main factors of variation in the Rhamnaceae gynoecium appear to be (1) the number of carpels and (2) their degree of union [8]. Our sampling ranged from one (e.g., *Rhamnidium elaeocarpum*) to four carpels (e.g., *Sarcomphalus joazeiro*), with varying degrees of union between the stigmatic branches. Nevertheless, the ontogenetic patterns observed here correspond to what has already been established for the family (e.g., [8,27,28,31]), such as the simultaneous emergence of the carpel primordia, their arrangement in a ring, and their horseshoe shape during the early stages.

### 3.3. The Relationship between Petals and Stamens in Rhamnaceae

Our results corroborate a floral ontogenetic pattern for Rhamnaceae, i.e., a close relationship between petals and stamens. The plastochron between petals and antepetalous stamens is very short, and the two organs show a union at the base from very early on. Thus, petals and stamens are organs that are always opposite and united at the base in Rhamnaceae. Even in the few Rhamnaceae species that lack petals (*Condalia* Cav., *Krugiodendron* Urb., and *Siegfriedia* C.A.Gardner, as well as some *Discaria*, *Gouania*, *Pomaderris* Labill., *Rhamnus*, *Reynosia*, and *Ventilago* Gaertn.) [5,25], the beginning of the development of these organs is marked by the formation of a functional stamen (internal) and a filamentous structure (external) (e.g., *Rhamnus japonica* Maxim) [3].

Some authors have postulated that petals and stamens originate from a common primordium in Rhamnaceae flowers (e.g., [3,6]). According to this interpretation, petals and stamens arise simultaneously from the tangential division of these common primordia. One possibility for this phenomenon is a progressive delay in petal development [26]. Thus, the petal primordia may be incorporated by the antepetalous stamen primordia, leading to the formation of a common primordium for both organs. This has been observed in several other families, such as Geraniaceae Juss., Plumbaginaceae Juss., and Primulaceae Batsch ex Borkh. [2,42]. The hypothesis of a common stamen–petal primordium would also explain the cases observed in *Sarcomphalus joazeiro* in which there was an increase in the number of stamens and petals. In this context, a radial division could occur before the tangential division of this common primordium, justifying the occurrence of twinned petals and stamens in an alternating position with the sepals.

Our data do not support the hypothesis of a common stamen–petal primordium for Rhamnaceae. Petals appear to emerge slightly earlier than the stamen primordia in *Hovenia dulcis*, *Rhamnidium elaeocarpum*, and *Sarcomphalus joazeiro*. This temporal difference has also been reported for *Oreoherzogia pumila* (Turra) W.Vent (=“*Rhamnus pumilus*”) [31], *Sarcomphalus mauritianus* (Lam.) Raf. (=“*Zizyphus mauritiana*” Lam.) [21], and *Ziziphus jujuba* Mill. [=“*Zizyphus sinensis*”] [31]. The main difficulty in recognizing the occurrence of a common primordium is that the division between petal and stamen is very rapid in most cases. Thus, it is important to intensify investigations on the origin of petals and stamens in Rhamnaceae. The example of Vitaceae is illustrative. This family was once considered a case of common stamen–petal primordia (e.g., [42]), but further studies on the group led to a refutation of this idea (see [43]). It is currently recognized that the stamens succeed the petals in Vitaceae—a similar condition to that observed here for Rhamnaceae.

The close ontogenetic relationship between petals and stamens in Rhamnaceae is also reflected in the functional relationship of these organs. Both organs commonly perform the same movements during anthesis; they are curved in bud, become erect during calyx opening, and become reflexed during anthesis [44]. This mechanism may be essential for reproductive success in these species, as exposed and accessible anthers are crucial for the more generalist pollination typical of Rhamnaceae. The studied petals (more so in *Colubrina glandulosa* but not in *Rhamnidium elaeocarpum*) form a hood-shaped structure that surrounds the anthers during most of the development stage inside the bud. The protection that these petals confer to the anthers may help to prevent pollen desiccation at anthesis [45].

### 3.4. Floral Structure of Rhamnaceae in the Context of Rosales

Rhamnaceae exhibit uniformity in ontogenetic patterns during floral development (e.g., [3,6,8,31,35] and the present study). However, their floral characteristics are markedly different from those of other Rosales.

A perigynous hypanthium is found in most Rosales but is lost in Barbeyaceae and urticalean rosids (Cannabaceae, Moraceae, Ulmaceae, Urticaceae). The perigynous hypanthium is conspicuous in Rhamnaceae and Rosaceae, with important ontogenetic implications in both families. The type of stamen development is more affected by the perigynous hypanthium in Rosaceae than in Rhamnaceae, especially in relation to the increase in the number of organs. The size and shape of the perigynous hypanthium in Rhamnaceae have a greater effect on the structure of the interstaminal nectary. A gynoecial hypanthium is only found in Moraceae, Rhamnaceae, and Rosaceae. It should be noted that inferior ovaries occur very rarely in Moraceae [46,47], whereas they are characteristic of some groups of Rosaceae, such as Maleae Small [19,48]. However, in Rhamnaceae, the occurrence and extent of a gynoecial hypanthium are very labile characteristics (see above).

The development of petals and antepetalous stamens is quite similar in Dirachmaceae and in Rhamnaceae; both types of organs alternate with the sepals and emerge at very short intervals (see [21]). Likewise, the meristic fluctuations of the androecium seem to follow the calyx merosity, independent of the floral merosity (e.g., pentamerous, tetramerous, or hexamerous). Among the urticalean rosids, the androecium exhibits only antesepalous stamens, commonly with only one or two stamens per flower [20]. The number of stamens in Rosaceae is more varied, with a predominance of the diplostemonous condition but also with the occurrence of a polystemonous or haplostemonous androecium [2]. In Barbeyaceae and Elaeagnaceae, groups supposedly close to Rhamnaceae, the androecium can be formed only by alternisepalous stamens (*Barbeya* Schweinf., *Elaeagnus*, *Hippophae*) or by antesepalous and alternisepalous stamens (*Barbeya*, *Shepherdia*) [49,50,51]. However, ontogenetic studies on these two families are not available for comparison with the other Rosales.

The presence of fewer carpels (2-3-4) than in the rest of the whorls merosity, as found in Rhamnaceae, is a common condition in rosids, including Pentapetalae [14]. Although such reductions may also be related to a decrease in the space available for carpel emergence, in the case of Rhamnaceae, such fluctuations do not seem to be correlated with calyx merosity. The degree of union between the carpels is a more variable characteristic among Rosales families. In the urticalean rosids, there is more than one carpel that composes the unilocular ovary (pseudomonomerous gynoecium [2,20]), and in Rosaceae, the carpels can be completely free or united [2,19]. Although Rhamnaceae have syncarpous ovaries, the styles vary greatly in their degree of union (see [8]). This variation in the development of more or less free style branches is similar to that found in urticalean rosids, especially among Moraceae and Urticaceae [20], although there is no evidence of a pseudomonomerous gynoecium in Rhamnaceae.

## 4. Material and Methods

### 4.1. Plants

Five species of five different genera were studied: *Colubrina glandulosa* var. *reitzii* (M.C. Johnst.) M.C.Johnst. (SPFR 17155), *Gouania virgata* Reissek (SPFR 17156), *Hovenia dulcis* Thunb. (SPFR 17157), *Rhamnidium elaeocarpum* Reissek (SPFR 17157), and *Sarcomphalus joazeiro* (Mart.) Hauenschild (RB 6544). Voucher specimens were deposited in the SPFR herbarium (Department of Biology, University of São Paulo, Ribeirão Preto Campus, SP, Brazil) and RB herbarium (Botanical Garden of Rio de Janeiro, RJ, Brazil).

Floral buds in different developmental stages and flowers were fixed in buffered formaldehyde (Lillie 1948 *apud* [52]) or FAA 50 (37% formalin, acetic acid, and alcohol) [53] for 24 h, stored in 70% alcohol, and prepared for surface (scanning electron microscopy (SEM)) and anatomical (light microscopy (LM)) exams.

### 4.2. Microscopy

For SEM exams, the samples were dehydrated in an ethanol series, critical-point-dried in a Bal-Tec CPD 030 dryer (Balzers, Liechtenstein), mounted on metal stubs, adhered to carbon adhesive tape, and covered with gold in a Bal-Tec SCD 050 sputter coater. Observations and illustrations were performed using a scanning electron microscope (Zeiss EVO-50, Cambridge, UK) at 15 kV.

After surface analysis (SEM), some stages were chosen for evaluation by light microscopy (LM). Samples were dehydrated in an ethanol series and embedded in histological resin [54], and transverse and longitudinal 3–3.5 μm thick sections were obtained with a rotary microtome (Leica RM 2245, Wetzlar, Germany). The sections were stained with toluidine blue in phosphate buffer (pH 5.8) [55] and mounted in water. Observations and illustrations were obtained using a Leica DM 4500 B LM connected to a Leica DFC 320 digital camera. Scales were determined under the same optical conditions.

## 5. Conclusions

The data obtained to date show that Rhamnaceae is a family with a very homogeneous floral development. Such stability is reflected in the emergence patterns of floral organs; the presence of intercalary growth, culminating in a perigynous hypanthium; the formation of keeled sepals; and the close relationship between petals and antepetalous stamens. Our data demonstrate how some modifications alter floral construction throughout development, such as petal formation (cucullate or not) and variation in ovary position. Just as the nectary is one of the most diverse and conspicuous features of the family and has a very similar development [7], flower development is, on the whole, curiously uniform in Rhamnaceae.

## Data Availability

Data sharing is not applicable to this article.

## References

[1] Jud N.A., Gandolfo M.A., Iglesias A., Wilf P. (2017). Flowering after disaster: Early Danian buckthorn (Rhamnaceae). PLoS ONE.

[2] de Craene R. (2022). Floral Diagrams.

[3] Bennek C. (1958). Die morphologische Beurteilung der Staub- und Blumenblätter der Rhamnaceen. Bot. Jahrb. Syst..

[4] Ronse De Craene L.P., Bull-Hereñu K. (2016). Obdiplostemony: The occurrence of a transitional stage linking robust flower configurations. Ann. Bot..

[5] Medan D., Schirarend C., Kubitzki K. (2004). Rhamnaceae. The Families and Genera of Vascular Plants.

[6] Beille L. (1901). Recherches sur le développement floral des Disciflores. Actes de la Société Linnéenne de Bordeaux.

[7] Ribeiro C.C., Marinho C.R., Mansano V.F., Teixeira S.P. (2022). The structural diversity of floral nectaries does not mean ontogenic diversity in Rhamnaceae species. Flora.

[8] Medan D., Leins P., Tucker S.C., Endress P.K. (1988). Gynoecium ontogenesis in the Rhamnaceae: A comparative study. Aspects of Floral Development.

[9] Prichard E.C. (1955). Morphological studies in Rhamnaceae. J. Elisha Mitchell Sci. Soc..

[10] Richardson J.E., Fay M.F., Cronk Q.C.B., Bowman D., Chase M.W. (2000). A phylogenetic analysis of Rhamnaceae using rbcL and *trnL-F* plastid DNA sequences. Am. J. Bot..

[11] Dahlgren R.M.T. (1980). A revised system of classification of the angiosperms. Bot. J. Linn. Soc..

[12] Takhtajan A.L. (1980). Outline of the classification of flowering plants (Magnoliophyta). Bot. Rev..

[13] Chase M.W., Christenhusz M.J.M., Fay M.F., Byng J.W., Judd W.S., Soltis D.E., Mabberley D.J., Sennikov A.N., Soltis P.S., The Angiosperm Phylogeny Group (2016). An update of the Angiosperm Phylogeny Group classification for the orders and families of flowering plants: APG IV. Bot. J. Linn. Soc..

[14] Soltis D.E., Soltis P.S., Endress P.K., Chase M.W., Manchester S.R., Judd W.S., Majure L.C., Mavrodiev E.V. (2018). Phylogeny and Evolution of the Angiosperms.

[15] Zhang S.-D., Soltis D.E., Yang Y., Li D.-Z., Yi T.-S. (2011). Multi-gene analysis provides a well-supported phylogeny of Rosales. Mol. Phylogenet. Evol..

[16] Thulin M., Bremer B., Richardson J.E., Niklasson J., Fay M.F., Chase M.W. (1998). Family relationships of the enigmatic rosid genera *Barbeya* and *Dirachma* from the Horn of Africa region. Plant Syst. Evol..

[17] Sun M., Naeem R., Su J.X., Cao Z.Y., Burleigh J.G., Soltis P.S., Soltis D.E., Chen Z.-D. (2016). Phylogeny of the Rosidae: A dense taxon sampling analysis. J. Syst. Evol..

[18] Stevens P.F. Angiosperm Phylogeny Website. http://www.mobot.org/MOBOT/research/APweb/.

[19] Kalkman C. (2004). Rosaceae. The Families and Genera of Vascular Plants.

[20] Teixeira S.P., Pedersoli G.D., Leme F.M., Leite V.G., Basso-Alves J.P., Demarco D. (2020). Development resolves the uncommon floral construction of urticalean rosids. Plant Ontogeny: Studies, Analyses and Evolutionary Implications.

[21] Ronse De Craene L.P., Miller A.G. (2004). Floral development and anatomy of *Dirachma socotrana*, (Dirachmaceae): A controversial member of the Rosales. Plant Syst. Evol..

[22] Wang G.-T., Shu J.-P., Jiang G.-B., Chen Y.-Q., Wang R.-J. (2021). Morphology and molecules support the new monotypic genus *Fenghwaia* (Rhamnaceae) from south China. PhytoKeys.

[23] Richardson J.E., Fay M.F., Cronk Q.C.B., Chase M.W. (2000). A revision of the tribal classification of Rhamnaceae. Kew Bull..

[24] Hauenschild F., Favre A., Michalak I., Muellner-Riehl A.N. (2018). The influence of the Gondwanan breakup on the biogeographic history of the ziziphoids (Rhamnaceae). J. Biogeogr..

[25] Hauenschild F., Matuszak S., Muellner-Riehl A.N., Favre A. (2016). Phylogenetic relationships within the cosmopolitan buckthorn family (Rhamnaceae) support the resurrection of *Sarcomphalus* and the description of *Pseudoziziphus* gen. nov. Taxon.

[26] Ronse De Craene L.P. (2018). Understanding the role of floral development in the evolution of angiosperm flowers: Clarifications from a historical and physico-dynamic perspective. J. Plant Res..

[27] Medan D., Hilger H.H. (1992). Comparative flower and fruit morphogenesis in *Colubrina* (Rhamnaceae) with special reference to C. asiatica. Am. J. Bot..

[28] Medan D. (1985). Fruit morphogenesis and seed dispersal in the Colletieae (Rhamnaceae). I. The genus Discaria. Bot. Jahr. Syst..

[29] Leins P., Erbar C. (2010). Flower and Fruit: Morphology, Ontogeny, Phylogeny, Function and Ecology.

[30] Kurzweil H., Kocyan A., Kull T., Arditti J. (2002). Ontogeny of orchid flowers. Orchid Biology VIII: Reviews and Perspectives.

[31] Payer J.-B. (1857). Traité d’Organogenie Comparee de la Fleur.

[32] Weberling F. (1989). Morphology of Flowers and Inflorescences.

[33] Schirarend C., Hoffmann P. (1993). Untersuchungen zur Blütenmorphologie der Gattung *Reynosia* Griseb. (Rhamnaceae). Flora.

[34] Ronse De Craene L.P. (2016). Meristic changes in flowering plants: How flowers play with numbers. Flora.

[35] Tel-Zur N., Schneider B. (2009). Floral biology of *Ziziphus mauritiana* (Rhamnaceae). Sex. Plant Reprod..

[36] Medan D. (1991). Reproductive phenology, pollination biology, and gynoecium development in *Discaria americana* (Rhamnaceae). N. Z. J. Bot..

[37] Zou F., Duan J., Xiong H., Yuan D., Zhang L., Niu G. (2017). Flower bud differentiation and development of ‘Jinsi No.4’ jujube (*Ziziphus jujuba* Mill.) in Hunan province of Southern China. Open Biotechnol. J..

[38] Soltis D.E., Fishbein M., Kuzoff R.K. (2003). Reevaluating the evolution of epigyny: Data from phylogenetics and floral ontogeny. Int. J. Plant Sci..

[39] Basso-Alves J.P., Goldenberg R., Teixeira S.P. (2017). The ontogenetic bases for variation in ovary position in Melastomataceae. Am. J. Bot..

[40] Evans R.C., Dickinson T.A. (2005). Floral ontogeny and morphology in *Gillenia* (“Spiraeoideae”) and subfamily Maloideae C. Weber (Rosaceae). Int. J. Plant Sci..

[41] Soltis D.E., Hufford L. (2002). Ovary position diversity in Saxifragaceae: Clarifying the homology of epigyny. Int. J. Plant Sci..

[42] Ronse De Craene L.P., Clinckemaillie D., Smets E.F. (1993). Stamen-petal complexes in Magnoliatae. Bulletin du Jardin Botanique National de Belgique.

[43] Gerrath J.M., Posluszny U., Melville L.H. (2015). Reproductive features of the Vitaceae. Taming the Wild Grape.

[44] Cerino M.C., Richard G.A., Torretta J.P., Gutiérrez H.F., Pensiero J.F. (2015). Reproductive biology of Ziziphus mistol Griseb. (Rhamnaceae), a wild fruit tree of saline environments. Flora.

[45] Medan D., Aagesen L. (1995). Comparative flower and fruit structure in the Colletieae (Rhamnaceae). Bot. Jahrb. Syst..

[46] Berg C.C. (1972). Olmedieae, Brosimeae (Moraceae). Flora Neotrop..

[47] Berg C.C. (2001). Moreae, Artocarpeae, and Dorstenia (Moraceae), with introductions to the family and Ficus and with additions and corrections to Flora Neotropica Monograph 7. Flora Neotrop..

[48] Xiang Y., Huang C.-H., Hu Y., Wen J., Li S., Yi T., Chen H., Xiang J., Ma H. (2016). Evolution of Rosaceae fruit types based on nuclear phylogeny in the context of geological times and genome duplication. Mol. Biol. Evol..

[49] Baillon H.E. (1872). Elaeagnaceae. The Natural History of Plants.

[50] Bartish I.V., Swenson U., Kubitzki K. (2004). Elaeagnaceae. The Families and Genera of Vascular Plants.

[51] Friis I., Kubitzki K., Rohwer J.G., Bittrich V. (1993). Barbeyaceae. The Families and Genera of Vascular Plants.

[52] Clark G. (1981). Staining Procedures.

[53] Johansen D.A. (1940). Plant Microtechnique.

[54] Gerrits P.O., Horobin R.W. (1991). The Application of Glycol Methacrylate in Histotechnology.

[55] O’Brien T.P., Feder N., McCully M.E. (1964). Polychromatic staining of plant cell walls by toluidine blue O. Protoplasma.

